# Lack of concentration-dependent local toxicity of highly concentrated (5%) versus conventional 0.5% bupivacaine following musculoskeletal surgery in a rat model

**DOI:** 10.1186/s40634-023-00591-2

**Published:** 2023-03-08

**Authors:** Jasper G. Steverink, Floris R. van Tol, Suzanne Bruins, Andre J. Smorenburg, Marianna A. Tryfonidou, Bas J. Oosterman, Marijke R. van Dijk, Jos Malda, Jorrit-Jan Verlaan

**Affiliations:** 1 Department of Orthopedic Surgery, University Medical Center Utrecht, Heidelberglaan 100, 3584CX, Utrecht, Netherlands; 2 SentryX B.V, Austerlitz, Netherlands; 3 Department of Clinical Sciences, Faculty of Veterinary Medicine, Utrecht University, Utrecht, Netherlands; 4 Department of Pathology, University Medical Center Utrecht, Utrecht, Netherlands

**Keywords:** Histopathology, Continuous wound infusion, Spinal surgery, Femoral surgery, Local anesthetics, Bupivacaine

## Abstract

**Purpose:**

Various sustained-release formulations incorporate high bupivacaine concentrations but data on local toxicity is lacking. This study explores local toxic effects of highly concentrated (5%) bupivacaine compared to clinically used concentrations in vivo following skeletal surgery, to assess the safety of sustained-release formulations with high bupivacaine concentrations.

**Methods:**

Sixteen rats underwent surgery, in which screws with catheters affixed were implanted in the spine or femur in a factorial experimental design, allowing single-shot or continuous 72 h local administration of 0.5%, 2.5% or 5.0% bupivacaine hydrochloride. During the 30-day follow-up, animal weight was recorded and blood samples were obtained. Implantation sites underwent histopathological scoring for muscle damage, inflammation, necrosis, periosteal reaction/thickening and osteoblast activity. Effects of bupivacaine concentration, administration mode and implantation site on local toxicity scores were analyzed.

**Results:**

Chi-squared tests for score frequencies revealed a concentration-dependent decrease in osteoblast count. Moreover, spinal screw implantation led to significantly more muscle fibrosis but less bone damage than femoral screw implantation, reflecting the more invasive muscle dissection and shorter drilling times related to the spinal procedure. No differences between bupivacaine administration modes regarding histological scoring or body weight changes were observed. Weight increased, while CK levels and leukocyte counts decreased significantly during follow-up, reflecting postoperative recovery. No significant differences in weight, leukocyte count and CK were found between interventional groups.

**Conclusion:**

This pilot study found limited concentration-dependent local tissue effects of bupivacaine solutions concentrated up to 5.0% following musculoskeletal surgery in the rat study population.

**Supplementary Information:**

The online version contains supplementary material available at 10.1186/s40634-023-00591-2.

## Introduction

Musculoskeletal surgeries are perceived as exceptionally painful [1]. Currently, postoperative pain is treated with multimodal analgesic protocols that aims to achieve synergistic effects between analgesics by targeting multiple pain pathways [2]. Frequently used analgesics in multimodal protocols include acetaminophen, non-steroidal anti-inflammatory drugs, local anesthetics and alpha-2-antagonists. Still, opioids fulfil a prominent role in analgesia after musculoskeletal surgery. Major drawbacks of opioid use are the frequency of adverse events and risk of dependence or addiction [3]. Opioid use and abuse are current societal challenges, with various (inter-)national initiatives being undertaken to limit opioid-related morbidity and mortality [4]. Despite treatment with opioids, poorly controlled postoperative pain is reported in up to 75% of patients [5]. The limited efficacy of postoperative analgesia and drawbacks of opioids leave a clear unmet need for effective and safe pain treatment following skeletal surgery.

Because sensory nerve fibers are abundant in skeletal tissue, local anesthetics have the potential to decrease opioid consumption following skeletal surgery as part of multimodal analgesic protocols [6]. With up to 8 hours of analgesia, bupivacaine provides the longest duration of action of all local anesthetics [7]. However, 8 hours of analgesia is insufficient for most (skeletal) surgeries, driving the development of various new sustained-release formulations of bupivacaine [8]. An advantage of local bupivacaine sustained-release formulations is their relatively high drug concentration at the target site, while systemic levels stay low [8]. However, multiple in vitro studies report concentration-dependent cytotoxicity of musculoskeletal cell types following bupivacaine exposure [9]. For example, bupivacaine at subclinical concentrations induces concentration-dependent decreases in mesenchymal stem cell proliferation, osteogenesis and wound healing in vitro, three important processes for successful recovery after musculoskeletal surgery [10]. The translatability of bupivacaine in vitro toxicity to the in vivo situation has recently been reviewed [9].

Clinically, bupivacaine is used in concentrations up to 0.5% (5 mg/mL), but novel bupivacaine sustained-release formulations use concentrations ranging from 1.33% to 10.5% [11, 12]. Such high concentrations are necessary to provide a sufficient dose of local anesthetic for extended durations of analgesia within manageable volumes. One such sustained-release formulation for use in skeletal surgery is currently under development by the authors, for co-implantation with screws [13]. However, local toxic effects of bupivacaine concentrations higher than used clinically are largely unknown, and knowledge of these effects is essential to understand the safety of bupivacaine sustained-release formulations [14]. To provide insight in the in vivo local toxicity of highly concentrated bupivacaine, this study infused high bupivacaine concentrations in a rat model following musculoskeletal surgery. We hypothesized that bupivacaine induced concentration-dependent toxic effects in musculoskeletal tissue after surgery.

## Methods

### Animals

National Animal Experiments Ethical Committee approval (AVD1150020197225) was obtained before the experiments. Study design and results were reported according to the ARRIVE guideline. Male Wistar rats (Envigo, HsdCpb:WU, 8 weeks old and weighing 250 g upon arrival) were used because of their extensive use in toxicology studies and bupivacaine pharmacokinetics comparable with humans [15, 16]. Upon arrival, rats were randomly picked from the box upon delivery to receive an ear-marking or not. A predefined schedule was in place to dictate the cage number and ear-marking of a rat, based on the sequence of picking. In each cage, a rat without ear-mark (blank) and a rat with a left ear-mark (left) were placed. The cages were numbered, resulting in animal codes in the format [cage number]-[blank OR left]. Using an online random sequence generator (www.randomizer.org), animal codes were matched to a treatment (consisting of administration method, administration location and bupivacaine concentration, e.g., D25S: Dosedump – 25 mg/mL - Spine). Rats were housed in pairs in standard cages with cage enrichment, a 12-hour light/dark cycle and air conditioning at 23 ± 2 °C with 60% humidity. Cages were cleaned at weekly intervals. Standard rodent chow and water were provided ad libitum. Animals were allowed 1 week of acclimatization prior to surgery, with daily handling by the researchers to reduce handling-related stress during experimental procedures. Pre-operative weights were recorded. Following surgery, animals were housed in solitary cages for 72 hours to avoid conflicts between animals, damage to the catheter and wound healing problems. After 72 hours, the catheter was cut at skin level to allow subcutaneous retraction of the catheter and healing of the skin. After the catheter was cut, the rats were housed in pairs again. Each individual rat served as experimental unit.

### Surgery

**Fig. 1:**
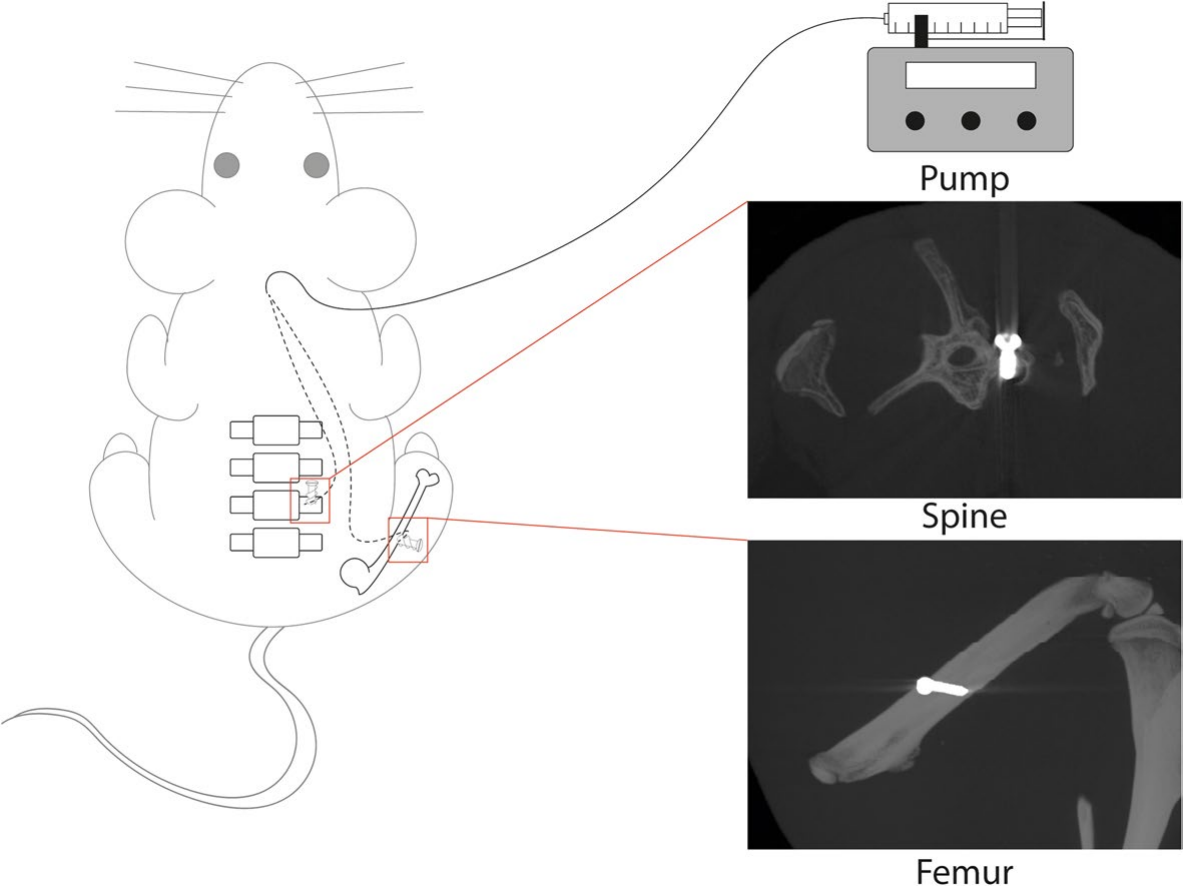
Schematic representation of experimental setup. Screws with a catheter attached were implanted in either the spine or femur. Catheters were then tunneled subcutaneously (dotted line) and exited between the shoulders. A pump was connected to the catheter to allow controlled infusion of bupivacaine solutions. Representative MicroCT images for the two surgical locations are shown

All rats received enrofloxacin (5 mg/kg), carprofen (5 mg/kg) and morphine (2.5 mg/kg) subcutaneously pre-operatively. To broaden generalizability, both a spinal and long bone (femur) application were performed. For spinal applications, rats were placed prone on a prewarmed surgery table under general anesthesia [17]. The iliac crest and spinous processes were identified and a midline incision was performed 1 cm cranially from the iliac crest. The paraspinal musculature was dissected laterally to expose the transverse processes of L4–5. A 0.8 mm hole was drilled in the transverse process and a stainless-steel screw (1*5 mm), attached via a poly(ε-caprolactone) connecting piece to a polyurethane (PU) catheter (internal diameter 0.6 mm, UNO, Zevenaar, Netherlands) was inserted, so that the catheter opening faced the bone surface (Fig. 1). Sterile saline solution was dripped next to the hole during drilling for cooling. The dead volume of catheters was pre-filled with the appropriate bupivacaine solution to ensure infusion of precise volumes. The catheter was tunneled subcutaneously towards an exit-point between the shoulders and fixed to the skin with a suture. Bupivacaine infusion was initiated after confirmed absence of motor deficits (i.e., following observed movement of tail and paws).

For femoral applications, rats were positioned laterally on the surgery table [18]. A longitudinal incision was performed lateral to the femur. The vastus lateralis muscle was separated from the femur by blunt dissection. A 0.8 mm hole was drilled in the femoral shaft and a stainless-steel screw attached to a catheter was inserted. The catheter was tunneled as in the spinal application.

### Test substance

Clinically used bupivacaine HCl 0.5% was obtained from Aurobindo Pharma (Baarn, Netherlands). Bupivacaine HCl powder was obtained from Siegfried (Evionnaz, Switzerland) and dissolved at 2.5% and 5.0% by a licensed veterinary pharmacy (Utrecht University, Netherlands). Bupivacaine solutions were delivered through the catheter to the surgical site. Potential adsorption of bupivacaine onto the inner lining of the catheter was tested by immersing 5 cm of catheter into a 5.0% bupivacaine solution for 72 h at 37 °C. Following rinsing to wash away any non-adsorbed drug, bupivacaine content was determined using a previously described UPLC method and expressed as μg drug absorbed per cm of catheter [19]. Catheters were attached to a gas chromatography syringe and pump, allowing precise infusion rates. Applying a factorial experimental design, rats underwent either spinal or femoral surgery and received 40 μL of bupivacaine 0.5% (clinically used control), 2.5%, or 5.0%, equating to 0.67, 3.3 and 6.7 mg/kg bodyweight, respectively. The bupivacaine solution was infused within a 10-second window (simulating dose-dumping) or in a sustained fashion (initial 50% of the total volume in the first 2 hours, followed by linear infusion of the remaining 50% over 70 hours), simulating burst release as displayed by various bupivacaine sustained-release formulations [8]. This factorial design yielded 12 combinations (Table 1). For every combination, a single rat was allocated. In case of termination of the experiment due to circumstances unrelated to the allocated treatment (e.g., technical failure, surgical complications), additional rats were available to repeat these treatments. To quantify correlations between each independent parameter (i.e., concentration, implantation site and infusion profile) and dependent parameters (i.e., histology scores, weight, leukocytes, CK), rats from the respective independent parameter subcategories were pooled.

**Table 1:** Treatment allocation, and histology subcategory scores for individual rats. 0 = None, 1 = Mild, 2 = Moderate, 3 = Severe, x = unable to score. Histology failed in one rat (rat 8 – D5S). DD = dose-dump administration, SUS = sustained administration

	Animal ID	Concentration (%)	Profile	Assessment	Necrosis	Fibrosis	Muscle					Inflammation	Bone damage	Periosteal reaction	Osteoblasts	Histiocytes
							Fibrosis	Atrophy	Calcifications	Necrosis	Inflammation					
Spine	L5S	0.5	SUS	Trajectory	0	1	1	2	2	0	0	0	2	2	2	0
				Screw head	0	1	1	1	0	0	0	0	2	2	2	0
	D5S	0.5	DD	Trajectory	Histology failed											
				Screw head												
	D25S	2.5	DD	Trajectory	0	3	3	1	0	0	1	0	3	2	2	1
				Screw head	0	1	1	1	0	0	0	0	2	2	1	0
	L25S	2.5	SUS	Trajectory	0	3	2	2	1	0	0	0	2	2	2	3
				Screw head	0	3	1	2	1	0	0	0	2	2	2	3
	D50S	5.0	DD	Trajectory	0	3	3	3	0	0	0	0	2	3	1	1
				Screw head	0	3	0	0	0	0	0	0	3	3	1	1
	L50S	5.0	SUS	Trajectory	0	0	3	3	0	0	0	0	3	2	1	2
				Screw head	0	0	2	1	0	0	0	0	2	1	1	0
Femur	L5F	0.5	SUS	Trajectory	0	1	0	0	0	0	0	0	x	x	x	3
				Screw head	0	3	1	1	0	0	0	0	3	2	2	1
	D5F	0.5	DD	Trajectory	0	0	0	0	0	0	0	1	x	x	x	x
				Screw head	0	2	1	0	0	0	1	3	3	3	1	3
	L25F	2.5	SUS	Trajectory	0	2	0	1	2	0	1	0	x	x	x	x
				Screw head	0	1	0	2	1	0	1	0	3	2	1	1
	D25F	2.5	DD	Trajectory	0	2	1	1	0	0	0	0	x	x	x	1
				Screw head	0	2	0	1	2	0	0	0	3	2	1	3
	L50F	5.0	SUS	Trajectory	0	3	0	1	0	0	0	0	x	x	x	2
				Screw head	0	1	0	1	0	0	0	0	3	2	1	1
	D50F	5.0	DD	Trajectory	0	1	1	1	0	0	0	0	x	x	x	x
				Screw head	0	1	1	3	0	0	0	0	3	1	1	1
	L25F	2.5	SUS	Trajectory	0	1	0	0	0	0	0	0	x	x	x	0
				Screw head	0	1	1	1	0	0	0	0	3	2	1	0

### Evaluation of well-being

Following surgery, animals were inspected for physical attributes such as appearance, wound healing and general condition. Well-being was scored daily by experienced caretakers. Weight was measured at predetermined intervals. Animals received carprofen and enrofloxacin (5 mg/kg) once daily until 72 hours after surgery. When the clinical condition of an animal caused concern, a veterinarian was consulted and action was taken (and documented) and/or monitoring was adjusted according to dictating circumstances.

### Systemic toxicity: blood and serum analysis

Blood was sampled from the tail vein at 1, 2, 4, 24 and 72 hours, 11, 18 and 30 days after surgery, and a volume of 100-200 μL was collected. Leukocyte counts as a marker for inflammation, and CK levels as a marker for muscle damage were quantified by a blinded veterinary diagnostic laboratory. Bupivacaine serum levels were measured using a commercially available ELISA kit (DEIA-XYL46, Creative Diagnostics, NY, USA).

### Local toxicity: histological analysis

Animals were euthanized 30 days after surgery by CO_2_ asphyxiation. Gross necropsy was performed according to Registry of Industrial Toxicology Animal data (RITA) and North American Control Animal Database (NACAD) guidelines [20]. Screw positioning and surrounding bone quality were imaged directly following euthanasia using computed tomography imaging (MicroCT: Quantum FX, Perkin Elmer, MA, USA). Implantation sites were then resected *en bloc* and stored in 4% buffered formaldehyde. To prevent metal oxidation, screws were removed prior to sample decalcification yet after tissue fixation. Thereafter, samples were decalcified (Rapid Decalcifying Solution, Klinipath, Netherlands) and embedded in paraffin. 4 μm sections were stained using hematoxylin (Haemaluin, Fisher Scientific, MA, USA) & eosin (Eosine G(Y), Merck, Germany) stain. Three to five sections were assessed per animal in two locations: at the level of the screw head and in the surgical trajectory. For every animal, two sections containing either the screw hole or trajectory, and visually most extensive damage were scored in a semi-quantitative fashion by a blinded board-certified pathologist using standardized terminology [21]. A Nikon Eclipse E900 microscope was used for imaging. All slides were screened for relevant tissue processes and cell types to be included for scoring. Next, the range in which these processes took place was determined on an ordinal scale (0 = absent, 1 = mild, 2 = moderate, 3 = severe). Included categories for scoring were: bone damage, fibrosis, presence of histiocytes, inflammation, necrosis, osteoblasts and periosteal reaction. Muscle tissue was separately scored for atrophy, calcifications, fibrosis, necrosis and inflammation. The density of polymorphonuclear cells was used to score inflammation.

### Statistical analysis

As this study aimed to provide a proof-of-principle of bupivacaine-induced toxicity after musculoskeletal surgery, no sample size calculations were performed. Statistical analysis was performed using Rstudio (Boston, USA). Data normality was assessed using Q-Q plots and a Shapiro-Wilk test. All continuous parameters were reported as means ± SD. Effect of bupivacaine concentration on local toxicity scores was analyzed using Chi-squared tests, comparing the frequency of category scores (0–3) between groups. In case of significance, Chi-squared tests were combined with a Bonferroni post-hoc test to correct for multiple comparisons. Effects of infusion profile and implantation site on local toxicity scores were analyzed using Chi-squared tests. Sensitivity analyses were performed by employing multivariable ordinal logistic regression analysis (Supplementary information). Weight, CK and leukocyte counts were analyzed as dependent parameters using mixed-effects models. Time since surgery, infusion profile, bupivacaine concentration, implantation site and any interactions between these factors were used as fixed effects, while also incorporating a random intercept for each individual rat and a random slope for time since surgery per individual rat. To determine significance, *p* < 0.05 was used.

## Results

### Evaluation of well-being and complications

Sixteen rats underwent surgery. One rat (L5F) died shortly after surgery because of excessive blood loss following femoral drilling and was replaced. Taking into account the blood loss during surgery and evident hypovolemic shock (e.g., pallor), the death was considered unrelated to drug exposure and the treatment was therefore repeated in another rat. All other animals recovered without incidents from surgery. In one rat (L25F), the catheter broke after 8 h of infusion, necessitating a reiteration of the experiment to ensure adequate bupivacaine exposure. Following surgery and bupivacaine administration, rat D50F displayed abnormal behavior: gagging, drooling, rhonchi, and tachypnea. The rat received atropine (0.1 mg/kg) treatment and recovered the day after surgery. Because a potential relationship between dose dumping of a high concentration of bupivacaine and these symptoms could not be excluded, the 5.0% bupivacaine dose-dump scenario for both a spinal and femoral administration were repeated in two rats (D50F and D50S). Repeat rats receiving the 5.0% bupivacaine dose-dump scenario did not undergo histological analysis. MicroCT imaging did not display signs of impaired bone healing in any rat (e.g., radiolucent areas around the screws). Furthermore, all screws were tightly anchored in the bone upon post-mortem retrieval. During gross examination following termination, petechia in the thymus were reported in two rats (L5S and L50S).

### Histological analysis of local toxicity

**Fig. 2:**
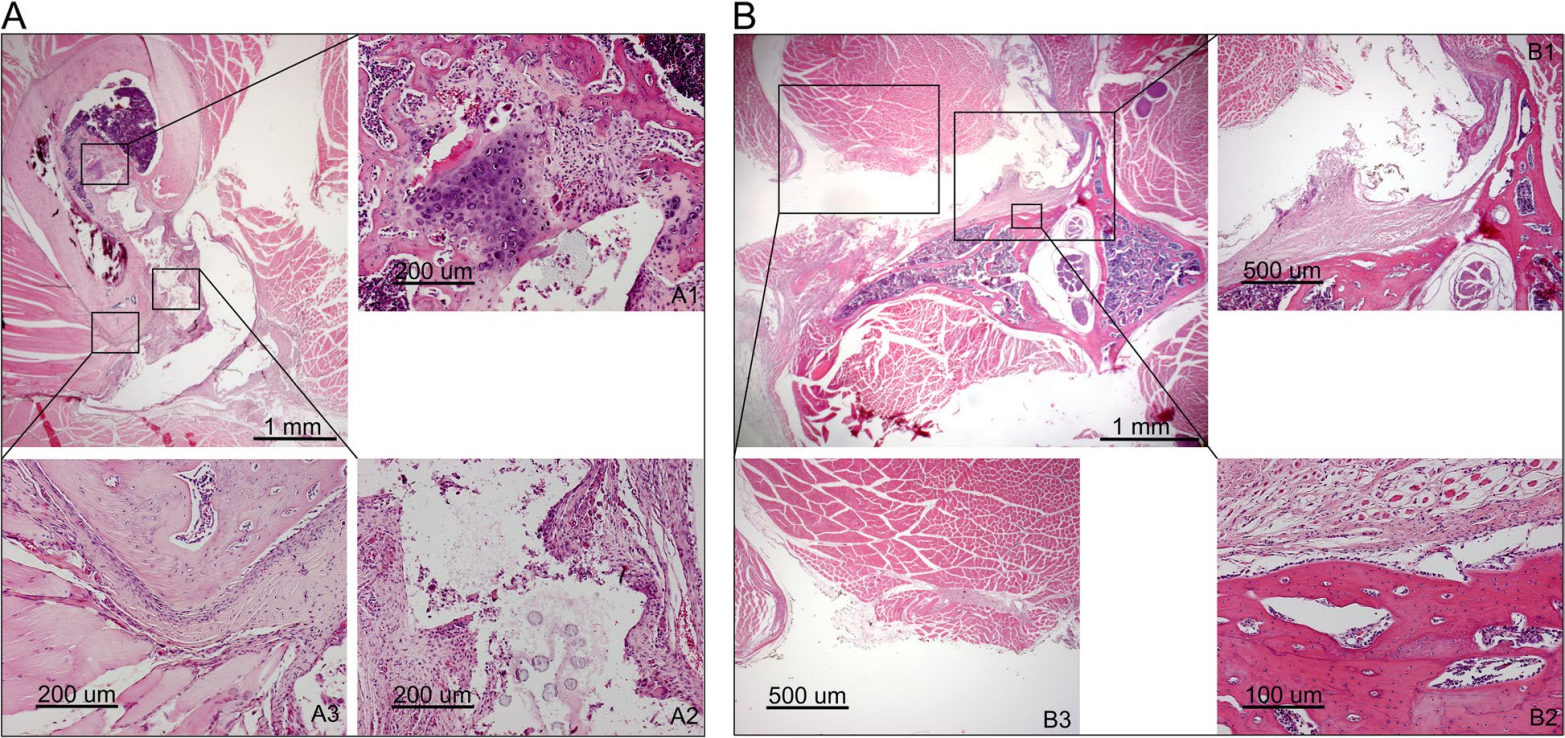
Representative histology images. **A** Hematoxylin and eosin staining of a femoral implantation site with cartilaginous new bone formation in the screw trajectory (A1), fibrous capsule formation around the screw head (A2) and periosteal thickening (A3). **B** Hematoxylin and eosin staining of a spinal implantation site with fibrous capsule formation around the screw head (B1), abundant osteoblasts lining the cortex (B2) and muscle fibrosis (B3)

Cartilaginous new bone formation around the screw, fibrosis, presence of histiocytes, periosteal thickening and varying degrees of inflammation were evident after femoral implantation (Fig. 2A). Following spinal implantation, periosteal reaction, muscle fibrosis, muscle atrophy, and presence of histiocytes were reported (Fig. 2B).

**Fig. 3:**
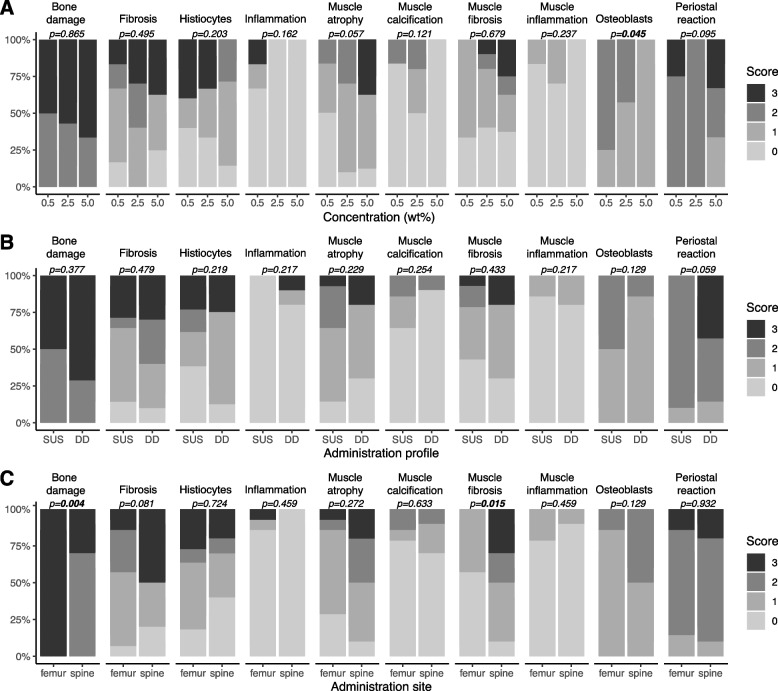
Bar charts displaying the frequency of histology scores per histological parameter as a function of **A**) bupivacaine concentration administered (wt%), **B**) bupivacaine administration profile (SUS – Sustained administration, DD – Dose Dump administration) and **C**) administration site (spine and femur). *P*-values obtained from Chi-squared tests are displayed. In case of a significant Chi-squared test and comparison between > 2 groups, a post-hoc Bonferroni testing was performed

Individual rat histology scores are displayed in Table 1. Preparation of histology slides failed in rat D5S, as the tissue had been damaged during resection and was no longer suitable for sectioning. Necrosis was absent in all of the samples studied. Fibrosis, histiocyte infiltration, inflammation, muscle atrophy, muscle calcification, muscle inflammation and periosteal reaction scores were not significantly influenced by bupivacaine concentration, administration site or administration profile (Fig. 3). Increasing bupivacaine concentration led to a significant decrease in osteoblast count (*p* = 0.045). Bonferroni post-hoc analysis revealed that the significant difference existed between the clinically used 0.5% concentration and the 5.0% concentration. A significant increase in bone damage (*p* = 0.004) and decrease in muscle fibrosis (*p* = 0.015) was observed when comparing femur with spine. Multivariable sensitivity analysis confirmed these findings (Supplementary).

### Secondary outcomes

**Fig. 4:**
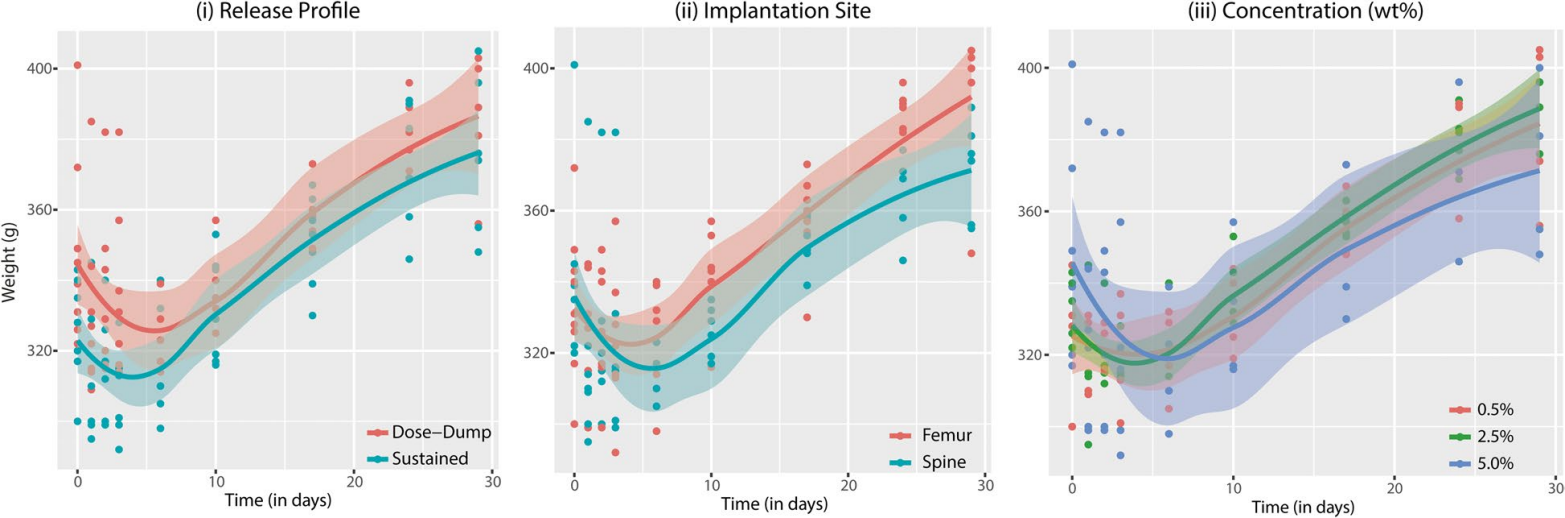
Body weight changes in rats undergoing surgery and infusion of bupivacaine. Median body weight and interquartile range (IQR) are shown. (i) Weight changes over time in rats receiving bupivacaine infusion in a dose-dump or sustained fashion. (ii) Weight changes over time in rats undergoing spinal or femoral catheter implantation and bupivacaine infusion. (iii) Weight changes over time in rats receiving infusion of 0.5%, 2.5%, or 5.0% bupivacaine HCl solution. T = 0 marks preoperative weight

Body weight was used as indicator for general animal well-being [22]. Following an expected initial decrease after surgery, weight increased steadily during postoperative recovery (Fig. 4). Rats undergoing dose-dump administration experienced a steeper decrease in postoperative weight, but recovered as fast as rats receiving continuous infusion. Postoperative leukocyte counts and CK levels are shown in Figs. S1 and S2, respectively. No delayed or disturbed wound healing was observed in any interventional group. Bupivacaine adsorption per cm of PU catheter was 2.5 ± 0.7 μg, when administering 5.0% bupivacaine. As 1 cm of catheter (diameter 0.6 mm) had a volume of 2.8 mm^3^, 14 cm of catheter contained 40 μL bupivacaine infused. This led to a potential loss of bupivacaine of 2.5 μg/cm * 14 cm = 35 μg. Taking the small adsorption values into account (35 μg on a total dose 2000 μg in the 5.0% bupivacaine group), this effect was presumed minimal.

Mixed-effect model analysis yielded no significant effects of implantation site, infusion profile or bupivacaine concentration on CK levels or leukocyte counts. Analysis of relations between fixed-effects displayed a positive interaction between time since surgery and weight (regression coefficient 0.91, *p* = 0.012) and negative interactions between time since surgery and CK (regression coefficient − 16.354, *p* < 0.001) and time since surgery and leukocyte counts (regression coefficient − 0.52, *p* = 0.001). Dose-dump bupivacaine administration led to serum concentrations ranging from below the assay detection limit of 5 ng/mL (when 0.5% bupivacaine was infused) to 170 ng/mL (infusion of 5.0% bupivacaine). All values were an order of magnitude below known systemic toxic values [23, 24]. Bupivacaine serum levels following sustained administration were below the detection limit of the ELISA kit, regardless of the infused concentration. Gross examination following euthanasia revealed petechiae in the thymus in two rats. This finding can be explained by the employed euthanasia method (CO_2_ asphyxiation), as presence of intrathoracic petechiae has been linked to asphyxiation in previous studies [25].

## Discussion

This study explored the local toxicity of highly concentrated bupivacaine in a skeletal surgery rat model, using the current clinical maximum concentration of 0.5% as a control. Bupivacaine is a frequently used local anesthetic in skeletal surgery, and has recently been applied in novel extended-release formulations at concentrations considerably higher than used in clinical practice [12, 26]. By employing a factorial design, the present study investigated the interaction between the administered concentration, implantation site and infusion profile. In line with in vitro reports on concentration-dependent inhibition of osteogenesis by bupivacaine, increasing bupivacaine concentrations significantly decreased osteoblast counts 30 days after spinal or femoral surgery [10]. However, no corresponding change in bone damage was found. Moreover, CT-imaging and screw explantation suggested undisturbed bone healing. No concentration-dependent muscle damage was observed, corresponding to previous animal studies showing regeneration of bupivacaine-induced muscle damage after 3 weeks [27]. Significantly more bone damage was observed in rats that underwent femoral surgery compared with spinal surgery. Likely, longer drilling times and subsequent heat generation in the much thicker cortex of the femur can explain this difference. In contrast, significantly more muscle fibrosis was observed in spinal surgery compared with the femur. This can be attributed to the more invasive nature of the spinal procedure: the paraspinal musculature was sharply dissected to expose the transverse processes, while blunt preparation between layers of the lateral vastus muscle was sufficient to obtain exposure of the femur. No significant differences between sustained and dose-dump administration were observed for any histological parameter. Altogether these findings agree with previous studies on 0.25% and 0.5% bupivacaine, indicating that the reported in vitro toxicity of bupivacaine does not translate to in vivo situations, where regenerative processes can take place [9, 28]. This conclusion now appears to be extendable to higher concentrations of bupivacaine.

No significant effects of bupivacaine concentration, implantation location or infusion profile on weight, CK or leukocyte counts were present in the mixed-effects model. Weight, CK and leukocyte counts correlated with the elapsed time since surgery, reflecting uncomplicated postoperative recovery. Despite the small study population and group size, the factorial experimental design allowed for exclusion of large effects of highly concentrated bupivacaine on skeletal local toxicity following surgery because of the inherent hidden replication, averaging the results over the levels of the other factors [29, 30]. Moreover, multivariable sensitivity analysis yielded results equal to univariable analysis (Supplementary). Univariable analyses were retained for clarity and readability. Reported bone healing rate in rats would have allowed assessment of effects of bupivacaine concentrations on bone healing, if present [31]. As all rats underwent surgery to receive bupivacaine infusion, the present results regarding the local toxicity of highly concentrated bupivacaine cannot simply be extrapolated to a non-surgical population. The addition of a control group receiving surgery but not bupivacaine would have further strengthened the conclusion that any local toxicity induced by bupivacaine is minor compared with the tissue damage through surgery, and its absence is a limitation of this study.

In conclusion, this preclinical study underlines the potential of newly developed sustained-release formulations of bupivacaine, as no major concentration-dependent local toxicity of high bupivacaine HCl concentrations was found following musculoskeletal surgery in the rat study population.

## Supplementary Information

**Additional file 1.**
