# Supplementary material for: Lack of concentration-dependent local toxicity of highly concentrated (5%) versus conventional 0.5% bupivacaine following musculoskeletal surgery in a rat model
Source: J Exp Orthop. 2023 Mar 8;10:21. doi: 10.1186/s40634-023-00591-2 (PMC9995632; doi:10.1186/s40634-023-00591-2)

**No increase in local toxicity of highly concentrated (5%) versus conventional 0.5% bupivacaine following musculoskeletal surgery in a rat model**

*Journal of Experimental Orthopaedics*

*Jasper G. Steverink^1,2^*, Floris R. van Tol, Suzanne Bruins, Andre J. Smorenburg, Marianna A. Tryfonidou, Bas J. Oosterman, Marijke R. van Dijk, Jos Malda, Jorrit-Jan Verlaan*

*Affiliations:*

1. Department of Orthopedic Surgery, University Medical Center Utrecht, Netherlands.
2. SentryX B.V., Austerlitz, Netherlands.

* Corresponding author. All correspondence to: Jasper Steverink, University Medical Center Utrecht, Department of Orthopedics, Heidelberglaan 100, 3584CX Utrecht. +31 88 755 5555. [J.g.steverink-4@umcutrecht.nl](mailto:J.g.steverink-4@umcutrecht.nl).

**Supplementary information – Sensitivity Analysis**

Following parametric testing of histology outcomes, a sensitivity analysis was performed by employing multivariable ordinal logistic regression analysis, setting histology subcategory as dependent variable and infusion profile (dose-dump vs sustained), bupivacaine concentration (0.5% vs 2.5% vs 5.0%) and implantation site (spine vs femur) as independent variables. For all statistical analysis, *p* < 0.05 was used to determine significance.

Multivariable logistic regression analysis revealed a significant effect of administration site on the severity of muscle fibrosis, corresponding to Chi squared test outcomes in the manuscript. Further, a significant effect of bupivacaine concentration on the severity of muscle atrophy was present. The significant effect of bupivacaine concentration on osteoblast count found in Chi squared testing was not reproduced in multivariable testing. No analysis for necrosis was performed. No other significant effects of concentration or infusion profile were observed in the multivariable logistic regression models.

| Multivariable ordinal logistic regression analysis | | Necrosis | | Fibrosis | | Inflammation | | Bone damage | | Periostal damage | | Osteoblasts | | Histiocytes | | Muscle fibrosis | | Muscle atrophy | | Muscle necrosis | | Muscle calcification | | Muscle inflammation | |
| --- | --- | --- | --- | --- | --- | --- | --- | --- | --- | --- | --- | --- | --- | --- | --- | --- | --- | --- | --- | --- | --- | --- | --- | --- | --- |
|  |  | Coeff (SE) | P-value | Coeff (SE) | P-value | Coeff (SE) | P-value | Coeff (SE) | P-value | Coeff (SE) | P-value | Coeff (SE) | P-value | Coeff (SE) | P-value | Coeff (SE) | P-value | Coeff (SE) | P-value | Coeff (SE) | P-value | Coeff (SE) | P-value | Coeff (SE) | P-value |
| Concentration (mg/mL) | 5 | Not analyzed, no cases of necrosis | | ref |  | ref |  | ref |  | ref |  | ref |  | ref |  | ref |  | ref |  | Not analyzed, no cases of muscle necrosis | | ref |  | ref |  |
|  | 25 |  |  | 0.996 (0.96) | 0.300 | -17.09 (72.5) | 0.814 | 5.902 (32.9) | 0.858 | 1.713 (1.52) | 0.261 | -15.56 (60.49) | 0.797 | -0.207 (1.13) | 0.855 | 0.148 (1.03) | 0.886 | 1.580 (1.04) | 0.128 |  |  | 1.373 (1.29) | 0.289 | 0.854 (1.34) | 0.525 |
|  | 50 |  |  | 0.024 (1.08) | 0.983 | -17.35 (79.1) | 0.827 | 7.089 (32.9) | 0.829 | -1.756 (1.67) | 0.293 | -38.45 (106.6) | 0.718 | 0.073 (1.10) | 0.947 | 0.800 (1.10) | 0.468 | 2.337 (0.89) | **0.0497** |  |  | -6.969 (24.5) | 0.776 | -7.210 (29.3) | 0.806 |
| Administration site | Femur |  |  | ref |  | ref |  | ref |  | ref |  | ref |  | ref |  | ref |  | ref |  |  |  | ref |  | ref |  |
|  | Spine |  |  | 0.649 (0.82) | 0.428 | -0.961 (70.81) | 0.989 | -14.9 (46.2) | 0.747 | 0.600 (1.17) | 0.609 | 22.71 (70.3) | 0.747 | -0.786 (0.85) | 0.353 | 3.551 (1.24) | **0.004** | 1.642 (0.89) | 0.065 |  |  | 0.420 (1.07) | 0.696 | -0.854 (1.34) | 0.525 |
| Administration profile | SUS |  |  | ref |  | ref |  | ref |  | ref |  | ref |  | ref |  | ref |  | ref |  |  |  | ref |  | ref |  |
|  | DD |  |  | 0.662 (0.80) | 0.408 | 16.55 (61.7) | 0.788 | 1.187 (1.60) | 0.459 | 2.340 (1.43) | 0.101 | -14.18 (49.0) | 0.772 | 0.548 (0.86) | 0.524 | 0.979 (0.858) | 0.254 | -0.852 (0.86) | 0.320 |  |  | -1.435 (1.31) | 0.272 | 0.673 (1.20) | 0.575 |

**Table S1** Multivariable ordinal logistic regression analysis of the effects of bupivacaine solution concentration, implantation site and infusion profile on cumulative and subdivisions of local toxicity scores. Significant p-values are presented in **bold**.

**Figure S1.** Leukocyte counts (in millions per mL) after surgery. T=0 marks the day of surgery. A) Leukocyte counts grouped by bupivacaine concentration. B) Leukocyte counts grouped by administration location. C) Leukocyte counts grouped by administration profile. The horizontal black bars represent the normal limits provided by the laboratory.

**
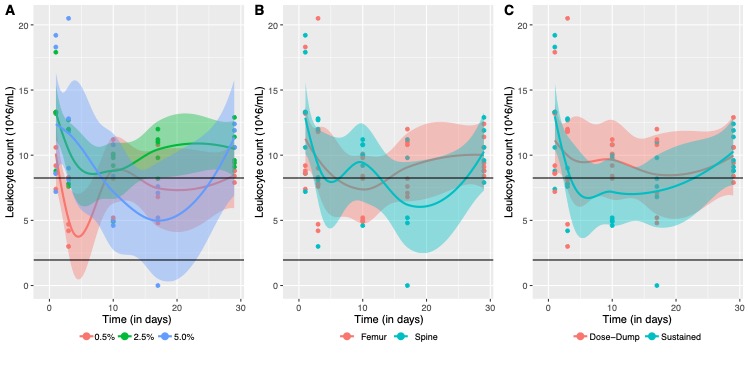
**

**Figure S2.** Creatine Kinase (CK) levels (in units per L) after surgery. T=0 marks the day of surgery. A) CK levels grouped by bupivacaine concentration. B) CK levels grouped by administration location. C) CK levels grouped by administration profile. The horizontal black bars represent the normal limits provided by the laboratory.


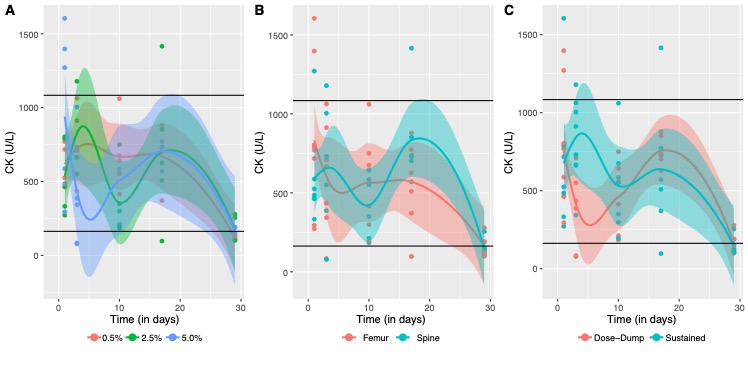

Supplement: Supplementary file 1 — Additional file 1. [file 40634_2023_591_MOESM1_ESM.docx]
